# The Influence of High-Frequency Envelope Information on Low-Frequency Vowel Identification in Noise

**DOI:** 10.1371/journal.pone.0145610

**Published:** 2016-01-05

**Authors:** Wiebke Schubotz, Thomas Brand, Birger Kollmeier, Stephan D. Ewert

**Affiliations:** Medizinische Physik and Cluster of Excellence Hearing4all, Universität Oldenburg, Oldenburg, Germany; University of Kent, UNITED KINGDOM

## Abstract

Vowel identification in noise using consonant-vowel-consonant (CVC) logatomes was used to investigate a possible interplay of speech information from different frequency regions. It was hypothesized that the periodicity conveyed by the temporal envelope of a high frequency stimulus can enhance the use of the information carried by auditory channels in the low-frequency region that share the same periodicity. It was further hypothesized that this acts as a strobe-like mechanism and would increase the signal-to-noise ratio for the voiced parts of the CVCs. In a first experiment, different high-frequency cues were provided to test this hypothesis, whereas a second experiment examined more closely the role of amplitude modulations and intact phase information within the high-frequency region (4–8 kHz). CVCs were either natural or vocoded speech (both limited to a low-pass cutoff-frequency of 2.5 kHz) and were presented in stationary 3-kHz low-pass filtered masking noise. The experimental results did not support the hypothesized use of periodicity information for aiding low-frequency perception.

## Introduction

Speech signals in general cover a wide range of frequencies and usually information across a wide frequency range is grouped to form a single auditory object [1]. However, in everyday life speech is rarely perceived in quiet, but in a masking noise and thus, not all parts of the spectro-temporal representation of the speech signal can contribute equally to speech perception. According to speech perception models such as the speech intelligibility index [2] or the glimpsing model [3], those parts of the representation that have large positive signal-to-noise ratios (SNRs) are most useful for speech perception. Therefore, any mechanism that increases the SNR can generally be assumed to improve the perception of masked speech stimuli. Such mechanisms can be external (e.g., a directional microphone in a mobile device or hearing aid) or internal in the auditory system, e.g. selection of appropriate auditory channels that carry specific speech cues. The current study aims at clarifying whether stimulus information derived from a high-frequency auditory channel can be used to enhance the identification of low-frequency speech sounds, precisely vowels, in a masking noise.

The information from the different frequency regions is thereby generally represented by different aspects of the filter output. Narrow auditory filters can extract the specific frequency components of a signal very accurately at low frequencies, e.g. resolve individual components of complex tones, whereas broader auditory filters at higher center frequencies extract information from the envelope of a signal only. Therefore the temporal representation of an analyzed signal can be very different for filters with different center frequencies (see [4]).

In case of the vowels tested in the current study, the human voice produces pulse trains with a periodicity that varies over time. In the frequency domain this corresponds to a complex tone with varying fundamental frequency F0 (inverse of the periodicity). Despite variations over time, periodicity and F0 can be regarded as quasi-stationary assuming a short-term analysis in the auditory system. When analyzed by narrow auditory filters in the low frequency-range, F0 is represented in a series of quasi-stationary frequency peaks (resolved harmonics). When analyzed by wider auditory filters in the high-frequency range, the periodicity is visible in the envelope of the filter output (unresolved harmonics). Therefore, this kind of periodicity information can be called F0-related information and occurs mostly in regions where the individual frequency components are unresolved [5]. Studies on the detection of pitch changes in complex tones [6–7] also suggest that periodicity information is encoded in the repetition rate of high-frequency temporal envelopes. For a wide-band signal such as speech, periodicity is thus correlated across different frequency regions, but is thought to be extracted with different mechanisms and represented by different aspects of the filter output. However, some studies (e.g. [5]) propose a single mechanism for the extraction of periodicity information from a speech signal across different frequencies.

The influence of F0 and resulting periodicity on speech perception is twofold. On the one hand, F0 and periodicity information can be used for the segregation of speech, on the other hand they also facilitate a combination of information across different frequency regions. Former studies [8–9] showed that e.g., the discrimination of two synthesized vowels and vowel identification is easier when the two presented stimuli do not share the same F0, while Broadbent and Ladefoged [10] showed that formants are grouped together if they share the same F0. Bregman et al. [11] stated that in addition to periodicity, temporal aspects are also important. They showed that congruent amplitude modulations across several frequency regions are fused and support the discrimination of two complex tones. Brokx and Nooteboom [12] and Bird and Darwin [13] reported that the F0 is also important for the intelligibility of longer speech tokens (short sentences). Brokx and Nooteboom [12] showed that intelligibility increased with pitch difference between target and interfering speech. In that study, listeners had to report the number of words they understood from (syntactically correct, but nonsense) target sentences when a constant difference in pitch between target and interfering speech was introduced by linear predictive coding (LPC). Bird and Darwin [13] investigated the mechanism by which the auditory system exploits F0 differences in separating two sentence-length utterances. They found that a common F0 is used to group components within the two sentences when the F0 of the individual utterances are more than 5 semitones apart. They suggested that for smaller differences, speech intelligibility is governed solely by factors in the low-frequency region (such as separate formants in the first formant region or individual harmonic components that are attributed to either the masker or the target sentence). This is also found in [14], where it is indicated that the discrimination of harmonic complex tones with different F0s relies primarily on low-frequency information, such as resolved lower harmonics.

In [15], detection and discrimination thresholds of low-frequency complex tones (designed as “stylized formants”) were found to improve significantly in the presence of an additional high-frequency cue band. This band provided information on temporal on- and offsets as well as periodicity of the low-frequency complex tone, but carried no other information. Discrimination improved even if the low-frequency complex tone and cue band were not in harmonic relation. Studies on coherence masking protection [16–17] found that certain cues (termed co-signal in [16] and [17]), although they alone did not provide direct information on the target signal, supported the perception of certain stimuli. In Gordon [17], it was shown that high-frequency vowel energy can provide such cue. In that study, listeners had to distinguish between the vowels /ɛ/ and /i/ that had different first formant energies (more than a critical band apart), whereas the high- frequency energy was identical for both vowels. Discrimination between both vowels increased, according to [17], because the first formant energy could be fused with the high-frequency vowel energy and enhanced the percept of the vowels. In [16], listeners had to identify synthesized vowels that consisted of a sine wave at a frequency corresponding to the first formant of the vowel /ɛ/ or /i/ and the co-signal, which was synthesized vowel energy corresponding to the second and third formant of the vowel /ɛ/. The co-signal was appropriate for either, /ɛ/ and /i/, and stimuli were only perceived as a certain vowel when sine wave and co-signal were presented together. It was found that identification thresholds decreased significantly due to the presence of the co-signal, suggesting that, although it was spectrally separated by more than a critical band from the sine wave, it contributed to the perceived sound. It was suggested in [16] that this was caused by auditory grouping of sine wave and co-signal due to the exploitation of regularities in the temporal pattern of the two. This finding persisted when the synthesized vowel energy was replaced by a complex tone that had the same amplitude modulation for all its components. However, identification thresholds only decreased when the co-signal was temporally aligned with the rest of the stimulus [16].

Based on results by [15–17] it can be hypothesized that periodicity information presented in a co-signal from a high-frequency region supports the perception of speech parts with the same periodicity in another frequency region. Such a hypothetical mechanism would be conceptually similar to the strobed integration stage as proposed by [18]. This describes a temporal integration stage that is sensitive to periodicity and stabilizes those structures in the neural responses to stimuli that share the same periodicity. The hypothesized mechanism could use F0-related temporal envelope information from a high-frequency region to support the perception of low-frequency components that share the same periodicity. Temporal peaks in the F0-related temporal envelope of the high-frequency part of the stimulus would define the “strobe points” that promote a certain periodicity. It is hypothesized that low-frequency channels with the same periodicity are selected and that the overall signal-to-noise ratio of those low-frequency channels is thus improved. The hypothesized mechanism would work best when the temporal envelope peaks and temporal fine structure information in the lower frequencies of the stimulus have a fixed phase relation across the frequency regions. This is the case for voiced parts of human speech consisting of pulse trains filtered by the vocal tract transfer function. In principle, this mechanism could also apply for any sound with quasi-periodic properties, such as voiced speech or music.

The current study examined whether F0-related temporal envelope information derived from high-frequency (4–8 kHz) channels can facilitate the identification of vowels in a masking noise in the low-frequency region below 2.5 kHz. It is, based on a strobe-like mechanism proposed in [18], and therefore constitutes a feasibility study. It is hypothesized that this mechanism works best in situations where speech is quasi-stationary (vowels), thus only a small portion of everyday speech is examined. The high-frequency periodicity information was provided in a high-frequency cue band with various configurations. The high-frequency cue band itself did not carry speech information that could be used when presented in isolation, but was thought to aid vowel identification.

In experiment 1, the experimental design of [15] was extended by using speech stimuli instead of complex tones and vowel identification instead of a psychoacoustic discrimination task. This was done to test if a hypothesized strobed integration, as proposed in [18], due to common periodicity [11, 17, 19] is in principle possible for stimuli that are similar to the vowels in natural speech since the research hypothesis cannot be tested with unmodified natural speech. Earlier studies [11, 15, 17] used stimuli that were not natural, e.g. complex tones or synthesized vowels. The current study used vowels in consonant-vowel-consonant (CVC) logatomes to investigate the proposed mechanism and to be comparable to earlier studies. Vowels were either generated with linear predictive coding (LPC) or by low-pass filtering intact speech material. Thus, the stimuli in the current study bridged the gap between stimuli that were not natural (complex tones) and natural speech. Modifications of the high-frequency cue band were tested to assess the role of temporal fine structure information from the high-frequency region as a possible co-signal, provided combined with the low-frequency masked CVCs.

In experiment 2, certain high-frequency speech cues (i.e., amplitude modulations and phase information) were presented in addition to the low-pass filtered logatomes or in isolation to test the hypothesis that these cues alone cannot lead to a substantial performance in vowel identification. If this was not the case, an improvement in vowel identification rates could be ascribed to those speech cues alone, instead of the periodicity information that is thought to be important for the strobed integration.

## Methods

### Ethics Statement

Written consent was obtained from each participant prior to the experiments. The experiments were approved by the local ethics committee of the University of Oldenburg.

### Subjects

Seven subjects, aged 25–32 years, participated in the first experiment. Six of them also participated in the second experiment. All listeners had an audiometric threshold of less than 20 dB HL or better at octave frequencies between 125 Hz and 8 kHz, except for one person who had 25 dB HL at 8 kHz. All listeners were naïve to the speech material and received an hourly compensation for their participation.

### Apparatus and procedure

A five-alternative forced choice vowel identification task (see [20]) was performed using a subset of the CVC logatomes of the Oldenburger Logatome Corpus (OLLO, [21]). Forty CVCs with a combination of eight consonants and five long vowels were used. The sampling rate of the logatomes was 16 kHz. The subjects had to identify the vowel in the CVC logatomes which were presented in random order over 40 trials. For every CVC, the five response alternatives were shown on the computer screen and had to be selected. Feedback was given to the listeners. The order of the response alternatives on the screen was randomized each time. Additionally, the order in which the experimental conditions were presented was randomized as well, excluding the case that the same condition appeared in successive trials. The experiment was done with three repetitions altogether. A shorter training test list with ten logatomes for each condition was presented to the subjects prior to each session of the actual measurement at a SNR of -14 dB. The signals were presented diotically at 65 dB SPL via Sennheiser HD 650 headphones in a double-walled, sound-attenuating booth. The stimuli were generated individually at runtime with Matlab (2011), using an alternative-forced-choice software package [22]. The headphones were calibrated on a Bruel&Kjaer 4134 artificial ear.

### Stimuli

The stimuli were modified versions of 40 CVCs (OLLO, [21]), spoken by the same male speaker (S02M). The logatomes were selected with eight consonants ([b], [d], [f], [g], [k], [p], [t], [z]) and five long vowels ([a:], [e:], [i:], [o:], [u:]). Their mean fundamental frequency was 131 Hz. The stimuli were presented in an unmodulated masking noise (ICRA 1 noise from [23]), low-pass filtered with an 8^th^-order Butterworth filter with a cutoff-frequency of 3 kHz in order to mask the low-frequency parts of the CVCs. The ICRA 1 noise was derived from English text (see [23]) read by a female speaker that was filtered in three analysis bands (low-pass filter at 800 Hz, band-pass filter between 800–2400 Hz, and high-pass filter at 2400 Hz). Each band had a white spectrum and all three were added up to form the resulting noise. The added signal was then high-pass filtered at 100 Hz to produce a male speech spectrum (find more details on the rationale of the ICRA noises and the manipulations in [23]). For the current study it was important that the noise had a spectrum that was similar to that of the target speech (male speaker) and masked frequencies below 3 kHz. A possible enhancement of periodicity in a low-frequency region would not be observable in quiet, but only in situations where lower frequencies are masked.

The measurements were performed for two fixed SNRs: -14 dB and -18 dB calculated from the low-frequency part only. The logatomes had a length of about 500 ms (the segment representing the vowel about 250 ms) and were placed in the middle of 1.5 seconds of masking noise. The exact stimuli setup however was different for both experiments.

## Experiment 1

### Detailed stimulus description

**Fig 1:**
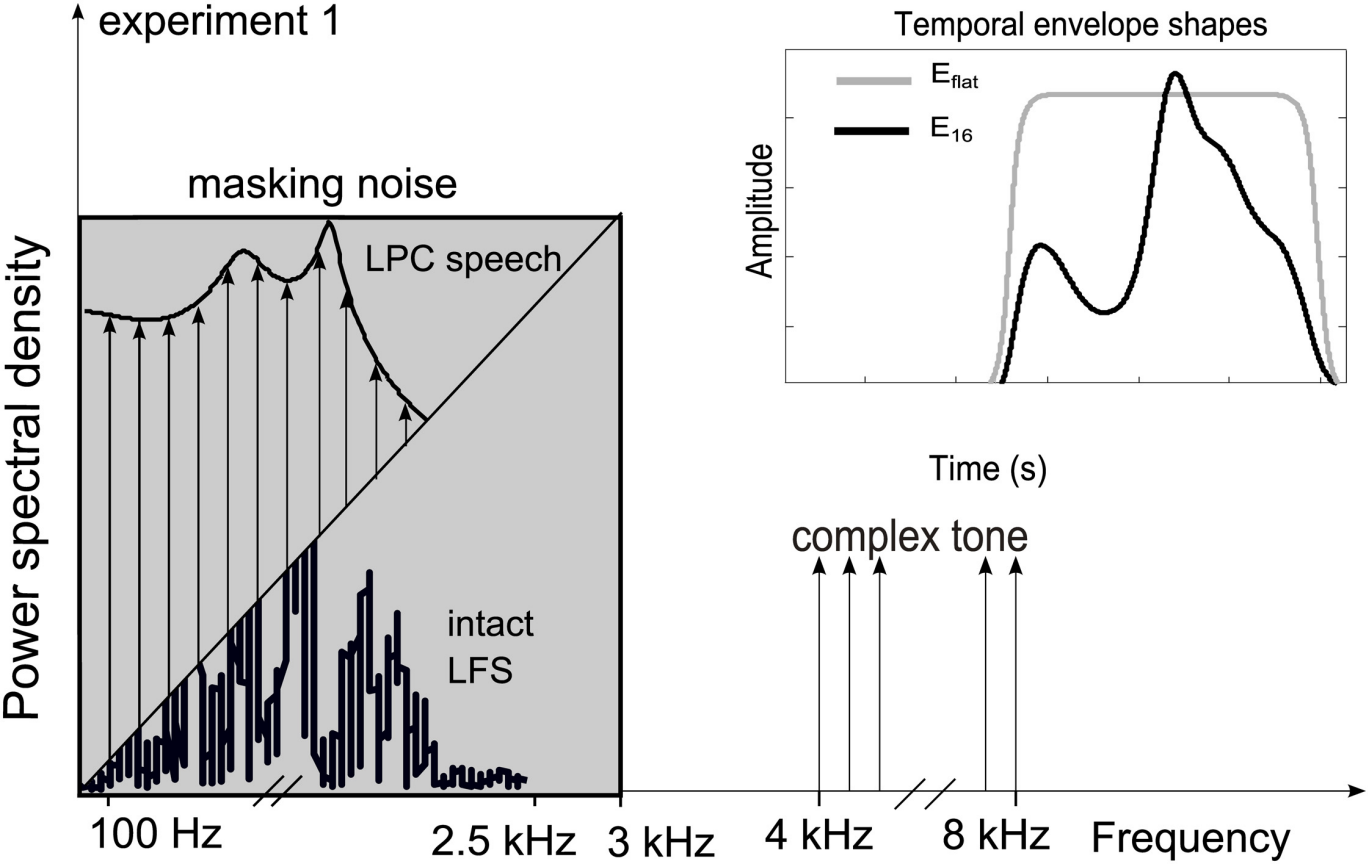
A schematic view of the experimental stimulus setup in experiment 1. Fig 1 shows the spectral properties (frequency regions of the complex tones, cutoff-frequency of the masker) of the low- and high-frequency part of the stimulus and the temporal shape of the high-frequency (HF) band envelopes of experiment 1. In this experiment, the low-frequency part of the stimulus was realized with either LPC-vocoded or intact, low-pass filtered speech (both limited to 2.5 kHz), as is schematically depicted in the left part of the figure. The flat envelope E_flat_ (gray line) encodes the on- and offset of the logatome, whereas the low-pass filtered envelope E_16_ (black line) ensures coherent amplitude modulations below 16 Hz in both frequency parts of the stimulus. Further high-frequency cues in experiment 1 are a 16-Hz low-pass filtered envelope (HFE_16_), phase information provided by infinite clipping (HFIC), and intact high-frequency speech (HFS), all derived from the region of 4–8 kHz from the intact logatome. All stimuli were presented in a low-pass filtered (cutoff-frequency was 3 kHz) stationary masking noise, indicated by the gray shaded area.

In the first experiment, the low-frequency part of the stimulus was either intact low-frequency speech (LFS) or a version of the logatome that was generated with linear predictive coding (LPC) as shown in Fig 1. LPC-vocoding was chosen to relate to findings from [11, 15, 19] where the stimuli were harmonic complex tones. The LFS was generated by low-pass filtering the intact speech with an 8^th^-order Butterworth filter with a cutoff-frequency of 2.5 kHz. This was chosen to sufficiently cover the region of the second formants (see [24]), which is especially important for the differentiation between [i:] and [u:]. The LPC speech was restricted to the same frequency region as the LFS. For the LPC-vocoding, the spectral envelope of the intact logatome was approximated by an all-pole filter with 20 coefficients in 100 ms time windows with a Levinson algorithm [25]. The envelope was imposed on a carrier that consisted of a harmonic complex tone (F0 = 100 Hz) with 25 components starting at 100 Hz that were all added in cosine phase. Thus, all natural F0 fluctuations of the speech stimuli were removed and a fixed F0 was set for the LPC synthesis, while the original spectral formant structure was maintained. Fixing the F0 led to a loss of voicing information (i.e., there is no distinction between voiced and unvoiced sounds), but this was irrelevant for the current study, since voicing information does not influence the identification of long vowels. Both low-frequency stimuli (LFS and LPC) were either presented alone in the masking background or in combination with an additional, simultaneous high-frequency cue band (HF band). The HF band consisted of a harmonic complex tone with 40 components (F0 = 100 Hz), starting at a frequency of 4 kHz. The cue band was multiplied in the time domain with two different temporal envelopes. The first condition provided a flat envelope (E_flat_), where on- and offset ramps were aligned with those of the LFS and LPC to allow for grouping due to common temporal on- and offsets across the two frequency regions. The second condition was a temporal envelope derived from the LFS. The envelope was extracted via the Hilbert transform from the LFS and low-pass filtered with zero-delay to 16 Hz by forward-backward filtering with a 2^nd^-order Butterworth filter. This low-pass filtered envelope (E_16_), allowed a transfer of the slowly varying amplitude fluctuations in the speech envelope from the low-frequency to the high-frequency region. Thus coherent amplitude fluctuations were provided in the frequency range below 2.5 kHz and in the frequency range from 4–8 kHz. Both HF band conditions were presented with LFS and LPC, as shown in Fig 1. Altogether, the experimental conditions were LFS, LFS-E_flat_, LFS-E_16_, LPC, LPC-E_flat_, and LPC-E_16_. The letters after the hyphen indicate that the HF bands were presented in addition to a certain low-frequency stimulus (LFS or LPC).

In combination with the LFS, three further HF band conditions were tested. While E_flat_ and E_16_ both transposed speech information originating from the low-frequency speech part of the logatome to the HF band, the additional conditions contained information from the intact high-frequency (4–8 kHz) speech part (HFS) of the logatome (indicated with the letters HF in the nomenclature of the experimental conditions). The intact HFS was generated by low-pass filtering the original speech token with an 8^th^-order Butterworth filter (cutoff-frequency was 8 kHz) and subsequent high-pass filtering with an 8^th^-order Butterworth filter (cutoff-frequency was 4 kHz). For the first additional condition, termed LFS-HFE_16_, the temporal envelope of the HFS was extracted via the Hilbert transform and then low-pass-filtered to 16 Hz by forward-backward filtering with a 2^nd^-order Butterworth filter. Thus, the envelope contained the slowly varying amplitude modulations that would naturally occur in the HFS. The HFE_16_ envelope was imposed onto the same complex tone as before. The second additional condition presented intact phase information (temporal fine structure) from the HFS together with the LFS. The phase information was extracted by determining the sign of the time signal (often referred to as infinite clipping), omitting amplitude fluctuations altogether. This condition is called infinite clipping (IC) condition, LFS-HFIC. The third additional condition was the intact HFS together with the LFS, called LFS-HFS. The different parts of the stimuli were set to the root mean square energy of the corresponding low- or high-frequency region of the original logatome to maintain the spectral energy distribution of the original speech token.

### Results

**Fig 2:**
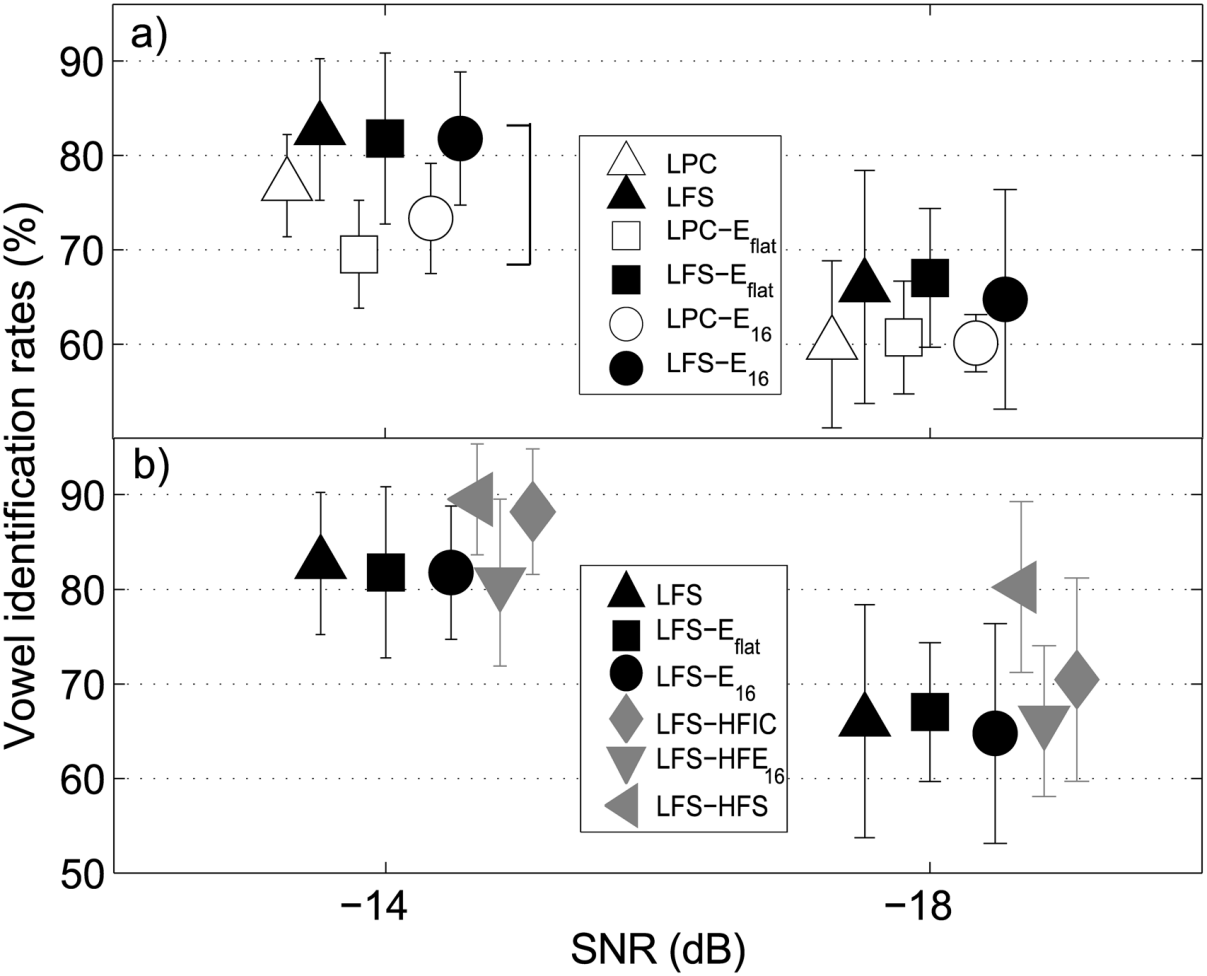
Vowel identification results of experiment 1. Mean vowel identification rates and corresponding standard deviations for the conditions in experiment 1. Panel a) shows the HF band conditions (E_flat_, E_16_) that were tested for both types of low-frequency design (LPC, LFS). Open symbols represent the rates that were measured when an LPC-vocoded logatome was presented in the low-frequency region of the stimulus. Filled symbols depict intact low-pass filtered logatomes in the low-frequency region of the stimulus. Stimuli where only low-frequency information was present are depicted with upward triangles. In panel b) the measured identification rates for all stimuli with LFS in the low-frequency part of the stimulus are presented. LFS-HFE_16_, LFS-HFIC, and LFS-HSF are depicted with gray symbols, while LFS, LFS-E_flat_, and LFS-E_16_ are replotted from panel a) with black symbols.

Fig 2 shows the mean vowel identification rates of experiment 1 and the corresponding standard deviations for both SNRs tested (-14 dB, left-hand side; -18 dB right-hand side). Panel a) shows those experimental conditions where LPC (open symbols) and LFS (filled symbols) were either presented alone (triangles) or in combination with E_flat_ (squares) or E_16_ (circles). Panel b) shows identification rates that were obtained with LFS and the various high-frequency cues indicated by the gray filled symbols. LFS, LFS-E_flat_, and LFS-E_16_ are replotted from panel a) as black filled symbols.

When comparing LFS and LPC only in Fig 2 panel a), LFS showed higher identification rates. This was also the case when E_flat_ and E_16_ were presented in addition to LFS and LPC alone. The vowel identification rates were in general about five (for -18 dB) to ten percent (for -14 dB) higher when LFS was presented instead of LPC speech. At -14 dB SNR the mean identification rate for the LFS alone was 83%, while it was 66% at -18 dB SNR.

The statistical analysis was not performed on the identification rates in percent correct, but on rationalized arcsine transformed units (rau). This transformation produces values close to the original percentage scores, but solves the problem of a limited range of values. A limited range can be a problem for statistical analysis when percentages appear that are close to the upper or lower ends of the scale and violate the assumption of a normal distribution. The rau transformation was performed using the equations (3) and (7) provided in [26] for the individual data from the listeners in all experimental conditions. The rationalized arcsine transformation maps the percent correct values on an open scale that is linear and additive, takes into account the binomial distribution assumption, and produces scores that can be interpreted like percentage. A three-way repeated measures analysis of variance (ANOVA) was performed on data from Fig 2a) with the main factors low-frequency part of the stimulus (LFS, LPC), HF band condition (no cue band, E_flat_, E_16_), and SNR (-14 dB, -18 dB). The analysis showed a significant main effect of SNR [F(1,6) = 1107.35, p<0.001] and low-frequency part of the stimulus [F(1,6) = 14.46, p<0.01]. Both values were Greenhouse-Geisser corrected. The HF band condition had no significant effect on vowel identification [F(2,12) = 2.32, p = 0.14]. Only the interaction of SNR and low-frequency part was significant [F(1,6) = 18.36, p = 0.005], all other interactions (SNR and HF band, low-frequency part and HF band, and the interaction of all three factors) were not significant.

In Fig 2b), the additional HFS-based information (gray symbols) showed an increase in performance for the intact HF speech (LFS-HFS, gray left-pointing triangle) and HFIC (gray diamonds), while performance stayed roughly the same for HFE_16_ (gray downward-pointing triangle). A two-way repeated measures ANOVA was conducted for the six conditions in Fig 2b), the main factors being SNR and HF band. The analysis showed a highly significant effect of the HF band [F(5,30) = 32.33, p<0.001] and the SNR [F(1,6) = 1673, p<0.001], the values for the SNR being Greenhouse-Geisser corrected. The interaction (Greenhouse-Geisser corrected) of both factors was not significant [F(1.83, 10.98) = 2.34, p = 0.14]. A post-hoc pairwise comparison (confidence level α = 0.05) using Bonferroni correction was performed to investigate the simple effect of HF band. It showed that identification scores for LFS-HFS differed significantly from all other conditions. Moreover, LFS-HFIC was significantly different from LFS-E_16_ and LFS-HFE_16_. Altogether, the presentation of F0-related information in addition to LFS improved vowel identification only in the LFS-HFIC and LFS-HFS condition. For LFS-HFS, the identification rates were generally about 10% higher than for all other LFS conditions.

## Experiment 2

### Rationale

Since the HFIC condition resulted in significantly improved vowel identification rates in experiment 1, the goal of experiment 2 was to examine possible explanations. Thus, the stimuli for experiment 2 were designed to specifically assess the role of phase information and amplitude modulations as a cue in the HF band. From experiment 1 it appears that phase information in the high-frequency region, conveyed in the HFIC is, as useful as intact HFS. A possible explanation is the use the phase information in the HF region. Another possible explanation is reconstructed envelope cues, as described in [27]. Ghitza [27] showed that if manipulated speech with a flat envelope is provided as the input of an auditory filter, envelope fluctuations of the original speech can be partly recovered at the filter output when the input still contains the original phase information. The output of an auditory filter is then not smooth, but shows similar modulations as if the original signal had been analyzed in that particular filter. Thus, the HFIC condition from experiment 1, which preserves the phase information, could convey envelope modulations at the output of auditory filters that are similar to intact HFS as a consequence of envelope reconstruction. Accordingly, not the high-frequency phase information itself, but reconstructed modulation cues could have improved the vowel identification in experiment 1.

### Detailed stimulus description

**Fig 3:**
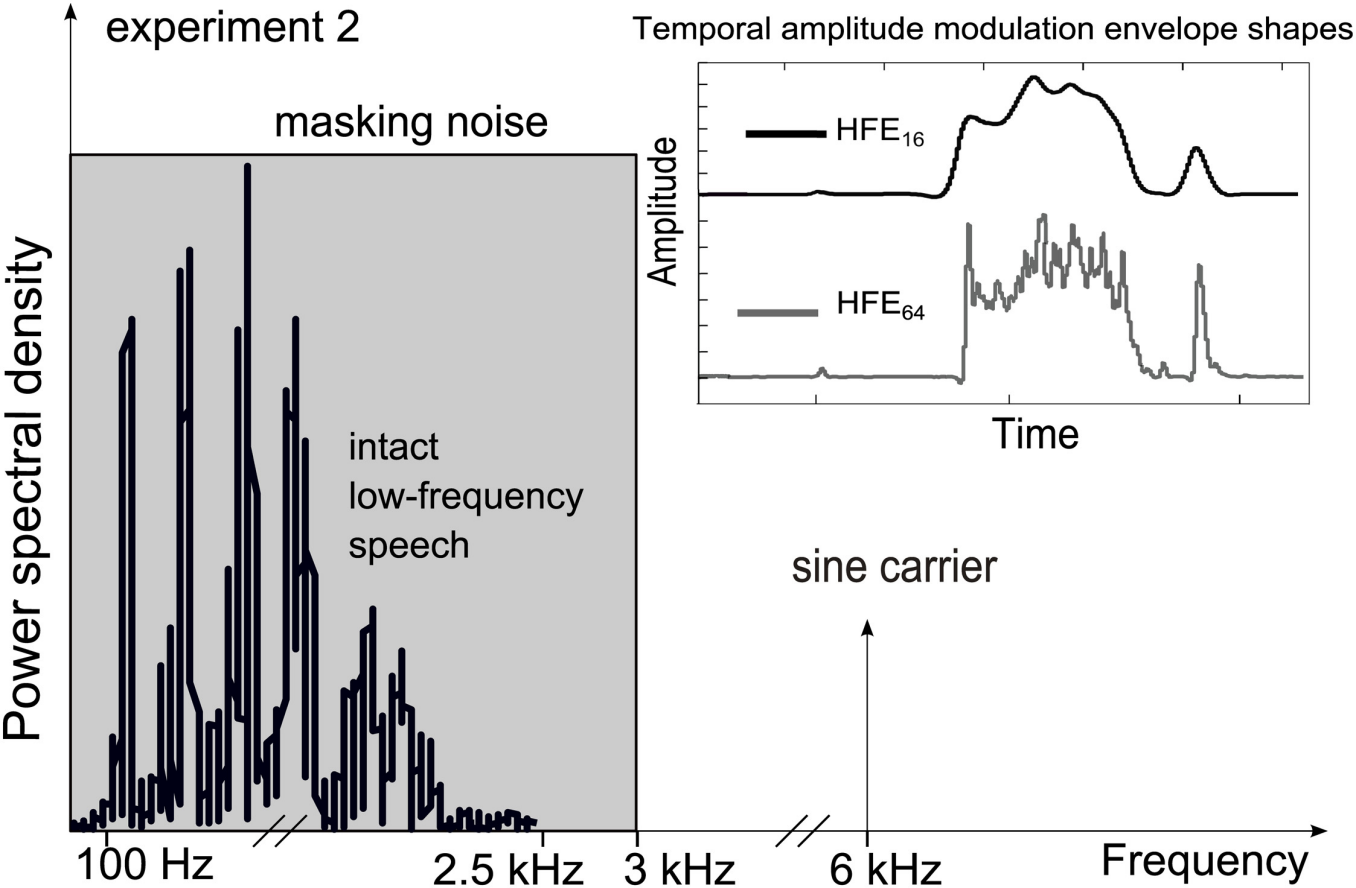
A schematic view of the experimental stimulus setup in experiment 2. Fig 3 shows the spectral setup of experiment 2. The HF band is replaced by a 6 kHz tone carrier, the low-frequency complex tone by intact low-pass filtered speech (LFS from experiment 1). The different low-pass filtered amplitude modulation envelopes (HFE_16_, HFE_64_) are shown in gray and black. Further high-frequency cues in experiment 2 are phase information provided by infinite clipping (HFIC from experiment 1), band-limited phase information provided by infinite clipping (HFIC_BP_), and intact high-frequency speech (HFS). Again, all stimuli were presented in stationary noise that was low-pass filtered at 3 kHz.

For the second experiment the logatomes were up-sampled to 96 kHz during signal manipulation, and then down-sampled and presented at 16 kHz, as this was the original sampling frequency. For this experiment the low-frequency part of the stimulus was LFS only, as shown in Fig 3. It was examined if a change in vowel identification occurred when the infinite clipping information is band-limited after phase extraction. Therefore, the HFIC condition in experiment 2 was slightly changed from the one in experiment 1: After the phase extraction from the HFS, a band-pass (BP) filter from 4 to 8 kHz was applied to the HFIC, in order to limit the frequency region of the phase fluctuations. This was achieved with a zero-delay 4^th^-order Butterworth filter. This experimental condition was called LFS-HFIC_BP_. To be able to compare this directly to the unfiltered condition, the HFIC condition was measured again in experiment 2 (LFS-HFIC).

The amplitude modulations from the high-frequency part of the logatome were provided by applying different low-pass filtered temporal envelopes of HFS on a 6-kHz sine carrier. The resulting spectrum of the modulated 6-kHz carrier was thus centered in the 4–8 kHz band. The use of a pure tone carrier ensured that the amplitude modulations at the output of an auditory filter most closely resembled the desired amplitude modulations and that the carrier phase did not carry any information. This 6-kHz sine carrier condition is indicated with HFE(S) in the following as it differed from the HFE setup of experiment 1, where the carrier was a complex tone.

The amplitude modulations in Fig 3 were generated by low-pass filtering the Hilbert envelope of the HFS of the logatome to 16 Hz and 64 Hz (LFS-HFE(S)_16_ and LFS-HFE(S)_64_). These frequencies were chosen with regard to [28], where it was suggested that energy modulations above 16 Hz are important in certain listening conditions and to allow for formant transitions within the CVC as those occur at modulation frequencies above 16 Hz. As in experiment 1, there was also the intact HFS presented as a high-frequency cue (LFS-HFS). All high-frequency cues were presented in addition to the LFS and also alone (without the LFS) in the masking noise. This was done to verify that improved vowel identification is caused by the presence of the additional high-frequency information (co-signal) that alone does not carry any valuable vowel information. As in experiment 1, the original energy distribution of the two frequency regions of the logatome was maintained.

### Results

**Fig 4:**
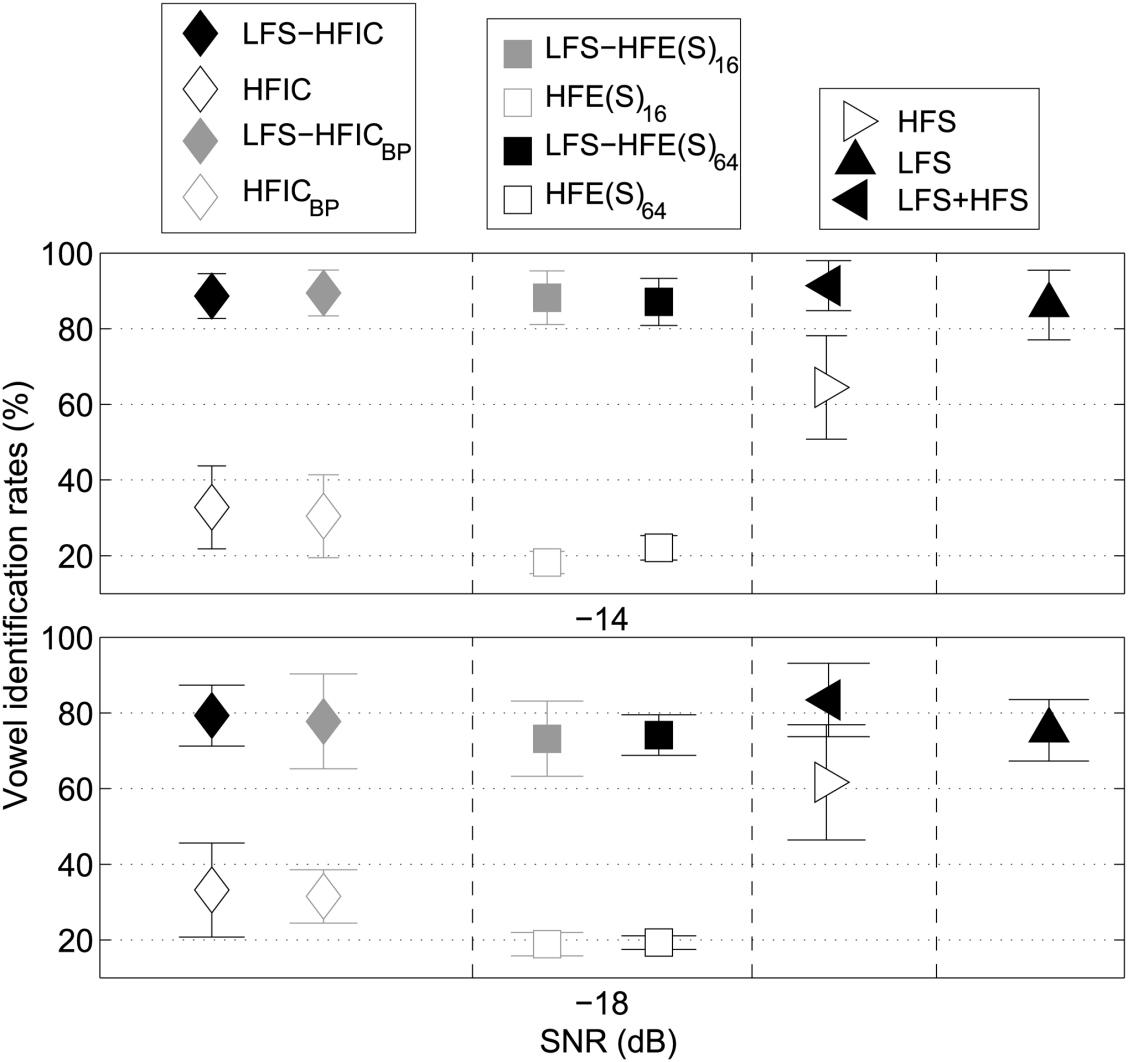
Vowel identification results of experiment 2. Mean identification rates and corresponding standard deviations for experiment 2. The upper panel shows the results for a SNR of -14 dB, the lower panel for a SNR of -18 dB. Filled symbols represent conditions where LFS and the high-frequency cues were presented, open symbols conditions where the high-frequency cues were presented in isolation. In contrast to experiment 1, the high-frequency envelopes were imposed onto a 6 kHz sine carrier instead of a complex tone. The left part of the figure shows the identification rates for the infinite clipping cues, the middle panel the rates for the amplitude modulation cues and the right panel the rates for intact speech cues. The rates for LFS alone are presented at the rightmost position as they serve as a reference for all other experimental conditions. As for experiment 1, all stimuli were presented in a stationary noise, low-pass filtered at 3 kHz.

Fig 4 shows the mean vowel identification rates for experiment 2 across the listeners together with the standard deviations. The upper panel shows the results for a SNR of -14 dB, the lower panel for -18 dB. The identification rates for HFIC conditions are depicted in the left part of the figure, identification rates for conditions with different cutoff-frequencies of the HF envelopes in the middle, and identification rates for intact speech in the right part of the figure. Filled symbols represent those experimental conditions, where the HF band was presented in addition to LFS and the low-frequency masking noise. Open symbols are for the respective HF bands alone in the low-frequency masking noise.

As for experiment 1, the pattern of results was similar for both SNRs. For the LFS-based conditions (filled symbols) identification rates were close to 90% at an SNR of -14 dB and about ten percent lower (about 80%) for the SNR of -18 dB. When the four HF bands (amplitude modulations and intact phase information) were presented in isolation, the identification rates were about chance level for the amplitude modulations and slightly higher for the presentation of IC and IC_BP_ at both SNRs. The rates for the presentation of the intact HFS alone were above 60% for both SNRs and thus substantially above chance level. When LFS alone was presented, rates were even higher, 88% (for -14 dB) and 77% (for -18 dB). The highest identification rates were reached when LFS and HFS were presented in combination and this was found for both SNRs. All six experimental conditions containing LFS were analyzed with a two-way repeated-measures ANOVA with the main factors SNR and HF band. A significant main effect of SNR [F(1, 5) = 196.98, p<0.001] and HF band [F(1.96, 9.8) = 7.07, p<0.001] was found, but not of the interaction between the two [F(5, 25) = 0.91, p = 0.49]. The values for SNR and HF band were Greenhouse-Geisser corrected. A post-hoc pairwise comparison (confidence level α = 0.05) using Bonferroni correction showed that identification rates for LFS-HFS were significantly higher than those for LFS-HFE(S)_16_, LFS-HFE(S)_64_, and LFS.

## General Discussion

### Testing the strobe-like mechanism

Although a strobe-like mechanism is supported by [11, 15, 16–17], it is unclear to what extent this can be assumed for vowel identification in speech signals. The current data does not support a benefit for vowel identification in noise by means of periodicity that is conveyed via high-frequency envelope information: When LPC-vocoded speech and F0-related temporal envelope periodicity information are presented at the same time, there is no improvement in vowel identification rates. The HF band conditions E_flat_ and E_16_ do not increase vowel identification performance although both share the same periodicity with the LPC-vocoded logatome. Moreover, they provide synchronous on- and offsets, which should enable grouping of the two sounds according to [29]. Furthermore, the E_16_ condition provides congruent amplitude fluctuations for both frequency regions, which should aid a grouping of the two sounds as according to [11, 19]. In contrast to the assumptions, no increase in vowel identification rates was observed and therefore it cannot be verified that a strobe-like mechanism as described in [18] occurs for the stimuli in the current study. This does not completely rule out such a mechanism, but it cannot be observed when periodicity information is presented in high-frequency envelope information. The results obtained in studies where stimuli were unnatural [10, 15–17] can therefore not easily be verified with stimuli that are closer to natural speech, such as CVCs.

A possible explanation for the lack of improvement opposed to earlier studies with non-speech stimuli could be the stimulus duration. In [15] is was stated that the absence of identification improvement for stimuli of about 500 ms or longer is caused by the long integration time in which within-channel information dominates across-frequency information. Stimuli in the current study are in total about 500 ms long, but the voiced part is about 250 ms long and thus a combination of across-frequency cues should in principle be possible. Stimulus duration alone is therefore not expected to be the main reason for the lack of identification improvement. Another possible explanation could be a part of the signal manipulation that could have caused a slight change in the formant structure of the vowels and thus vowel confusion: The spectral envelope used in the LPC-vocoding is derived from the original logatome with F0 = 130 Hz and is imposed on a complex tone of F0 = 100 Hz, which constitutes a typical fundamental frequency for male speakers [30]. This could have caused a slight shift of formants in the LPC-speech in the low-frequency range, but is unlikely to be the reason for a complete lack of identification improvement, because the formants should be analyzed in the same auditory filter as before.

Besides investigating the percent correct values, confusion matrices can be additionally assessed for each experimental condition (provided in S1 Fig for experiment 1). The confusion matrices suggest that the lack of vowel identification improvement is most probably caused by classification errors of certain vowels. Largest identification rates occur on the main diagonal and confusions only in submatrices. For the better SNR the [e:] is mistaken for [i:] and vice versa, and [o:] is mistaken for [u:] and vice versa, while all other possible confusions are negligible. This pattern smears out for the worse SNR, but still, confusions are largest for the submatrices [e:,i:], and [o:,u:]. Recognition of the vowel [a:] is very good throughout all experimental conditions, presumably because [a:] has no similar counterpart in the vowels tested in this study. This is because its position in the vocal triangle is far apart from the other vowels [31].

### The influence of intact low-frequency speech information

Examining identification rates when envelope information is presented in addition to LFS (instead of LPC-vocoded speech) does not investigate a possible strobing mechanism, but clarifies the role of the LFS itself. When LFS was used instead of LPC-vocoded speech, vowel identification rates increase significantly. This is in line with findings from [32–34] on simulated combined acoustic and electrical hearing, stating that additional low-frequency information leads to significant improvements in word intelligibility performance and is greater as the cutoff-frequency increases. Moreover, [32] showed that the presence of low-pass filtered intact speech at one ear enhances speech perception greatly, even if other low-frequency cues are presented at the other ear. This suggests a substantial influence of intact low-frequency speech to speech perception as is found in experiment 1 and experiment 2. The stimuli in the current study (intact low-frequency speech in addition to a complex tone) are similar to stimuli used in [32, 34], although the low-frequency region for those studies has a lower cutoff-frequency. Thus, findings from the current study can in principle be compared to those on combined hearing.

In experiment 1, no increase in vowel identification rates is observed when the E_flat_, E_16_ or HFE_16_ cue bands are presented in addition to LFS. The HF cue band with its fixed F0 conveys “false” F0 information in the HF region, producing a mismatch of periodicity for the two frequency regions. According to [12],”false” F0 in the high-frequency range can easily be rejected and thus LFS alone should suffice for the vowel identification observed. This is confirmed, because the identification rates are similar compared to LFS alone. When E_16_ is presented, vowel identification should increase due to grouping of congruent amplitude modulations [11, 19], but this is not observed. Again, low-frequency information (such as F0 fluctuations or formant transitions) seems to suffice for vowel identification in stimuli that are similar to natural speech. This is in line with findings from [35–36], stating that normal-hearing listeners rely primarily on resolved lower order harmonics when they segregate two concurring sounds. Moreover, the pattern of vowel confusions also suggests that most information on the vowels is already present in the LFS. Confusion matrices for experiment 2 (provided in S2 Fig) show that if LFS information is present in the stimuli, vowel identification is generally good. As for experiment 1, confusions appear only in sub-matrices for [e:, i:] and [o:, u:] and the vowel [a:] is robust towards confusion. If only a HF cue is presented, confusions are randomly distributed across the entire confusion matrix, confirming that the HF cue alone does not provide any substantial information on the vowel. The only exception is the vowel [i:] that shows larger identification rates than all other vowels for the condition HFIC and HFIC_BP_. Throughout experiment 2 there are hardly any confusions for [i:] and [u:] when LFS is present in addition to a HF cue, which means that information on the second formant is present to allow a distinction between both.

Data from both experiments show that HFS significantly improves vowel identification when combined with LFS. In experiment 2, HFS alone yields rates of about 60% correct, which is lower than the rates for LFS alone, but substantially above chance level. LFS alone leads to identification rates of about 86% correct for -14 dB (75% for -18 dB) and is increased by 6–8% when the combination of LFS and HFS is presented to the listeners. Combined intact speech results also in significantly higher identification results for experiment 1. This finding is similar to [37], where the influence of spectral slits on sentence intelligibility was investigated. The study states that even if intelligibility is reduced for single, narrow frequency bands, it rises tremendously when these are combined. The effect found in the current study is not as large as in [37], but still noteworthy. The maximally expected improvement under the assumption of optimal combination of independent information in LFS and HFS can be calculated using the combined error rates (see [38] for details). The error rates for HFS and LFS alone are 0.356 and 0.138, and 0.383 and 0.246 at -14 dB and -18 dB, respectively. The maximally expected identification rates for HFS and LFS in combination, as calculated from multiplication of the error rates for HFS and LFS alone, are 95.1% for -14 dB and 90.6% for -18 dB. The measured rates are 91.38% and 83.47%, which is less than the maximally expected rates, but still substantial, regarding the overall high identification rates.

### Possible use of phase information from the high-frequency region

Somewhat unexpectedly, the presentation of intact phase information (HFIC) in the HF band improves vowel identification significantly for experiment 1. There are two possible explanations for this improvement, the reconstruction of envelope fluctuations according to [27] and the use of temporal fine structure information in the high-frequency region above 4 kHz. If the improvement was caused by envelope reconstruction, conditions in experiment 2, where the corresponding amplitude modulations were provided as a HF cue, should have substantially improved vowel identification. However, this is not the case. The lack of identification improvement weakens the hypothesis that the LFS-HFIC condition is helpful because of reconstructed envelope cues. To rule out this possibility, subsequent measurements should be conducted in which amplitude modulations are presented over a smaller range of vocoder bands (e.g. one or more 1 ERB filters), instead of one broad filter range as done in the current study. This would allow a closer assessment of the frequency range in which recovered modulations are eventually helpful.

Results of experiment 1 leave the use of temporal fine structure as a possible explanation for the improvement of vowel identification. In contrast to literature like [39] or [40], reporting that phase-locking of the auditory nerve fibers limits the direct extraction to frequencies of 1–2 kHz, studies [41–43] report that the auditory system could have access to fine structure cues above 3 kHz. So far, it is under debate at which frequency a transition occurs from a direct extraction of the phase information (possibly at lower frequencies) to a place mechanism (possibly at higher frequencies). But these studies [41–43] show that a direct extraction is robust up to 6 kHz and indeed possible for even higher frequencies up to 8 kHz. This indicates that correlated phase information across frequencies, provided in the HFIC condition, could have caused the improved vowel identification rates in the current study, even if the phase information is band-limited as for the HFIC_BP_ condition.

### Limitations of the current study

Considering the overall identification rates in experiment 2, there is a general trend towards higher identification rates for conditions that were also measured in experiment 1 (LFS, LFS-IC, and LFS-HFS). This might be caused by training effects of the participants or by general ceiling effects, due to the amount of LFS information that is present in the stimuli. Listeners were naïve to the target material for experiment 1, however, six of the seven listeners also participated in experiment 2, which might have led to a training effect in the second experiment. On the other hand, experiment 2 was a follow-up study that took place half a year after the first experiment and thus, it is questionable to what extent listeners could rely on knowledge from the first experiment. Taken together, it is not obvious that the results from experiment 2 would differ much when a new set of listeners was recruited. Thus, further studies should be performed at slightly lower SNRs to reduce the ceiling effects. Generally, vowel identification rates are high for all conditions that include intact LFS information. A possible reason could be that the cutoff-frequency of 2.5 kHz for the low-frequency region of the stimuli is chosen too high and that therefore, vowel identification in both experiments is ruled mostly by the LFS alone. But this cutoff-frequency is chosen to allow a distinction between [i:] and [u:] which depends on the second formant of both vowels. Moreover, the low-frequency part of the logatome is masked by the background noise, so low-frequency information is not easily accessible.

Another possible reason for the lack of identification improvement might be the presentation of the stimuli in a stationary background noise. Studies, such as [33] and [44] show that normal-hearing listeners benefit from temporal envelope information only when the masker provides temporal gaps or is an interfering talker [45]. This is most probably caused by the ability of normal-hearing listeners to exploit the gaps in a masker and this leads to a release from masking in fluctuating maskers. Regarding [45], this effect is not as pronounced or even missing for hearing-impaired listeners or cochlea implant users. For the current study, however, a presentation in a stationary background is chosen, to be comparable to studies like [15] and [17] that use a stationary background noise, and to prevent the vowel from being unmasked: the logatomes of the current study are so short that the entire logatome could randomly fall in a gap of a fluctuating masker, reducing the low-frequency masking effect and probably strongly increasing variability in the data. But, it can be hypothesized that vowel identification with a similar setup should increase when measurement are done in a fluctuating masker that provide silent intervals in which the information from both frequency regions can be optimally combined.

## Conclusions

1. Vowel identification in CVC-logatomes in a stationary masking noise is improved for low-pass filtered speech when compared to LPC-vocoded speech limited to the same frequency range.
2. Findings on the improvement of identification of “stylized formants” in [15] could not be reproduced directly for signals that are closer to real speech than complex tones or synthesized vowels. The presentation of a high-frequency band with common periodicity, on- and offsets, and temporal envelope shape in addition to a complex tone has no effect on vowel identification.
3. The results do not support a hypothesized strobe-like mechanism that uses common periodicity information across frequency bands. With the current data it could not be verified that F0-related temporal envelope information, providing such periodicity information, aids the enhancement of frequency channels with the same periodicity in a low-frequency region. This does not rule out the existence of such mechanism, but it cannot be verified with stimuli chosen in the current study.
4. A significant improvement is observed in experiment 1 when the high-frequency band contains the intact phase information (HFIC condition) of the speech signal in that frequency band. The presentation of amplitude modulation cues in experiment 2 does not indicate that this improvement is caused by recovered amplitude modulation cues according to [27]. This leaves the use of temporal fine structure in the high-frequency region as a possible explanation for the vowel identification improvement in experiment 1.
5. Significant identification improvement is observed in experiment 1 when intact high-frequency speech is presented as a high-frequency cue in addition to low-frequency speech. In experiment 2 the intact high-frequency speech leads to a significant improvement in identification rates compared to other high-frequency cues. However, there is no improvement for most high-frequency cues in experiment 2, indicating that vowel identification is possibly ruled by the information in the low-frequency region of the stimuli.

## Supporting Information

S1 Fig Confusion matrices for the conditions in experiment 1. Matrices for the higher SNR are shown in the first two rows, those for the lower SNR in the last two rows. The rows indicate if LPC or LFS was used as a low-frequency part of the stimulus, columns indicate the type of high-frequency cue band that was presented. The color shading represents the identification rates. Black indicates perfect identification and white indicates no correct identification. The label on the left side of the matrix denotes those vowels that were presented to the listeners, the label on the upper side of the matrix denotes the vowels that were identified by the listeners. The numbers in the matrices correspond to the percentage of this certain confusion.
(PDF)

S2 Fig Confusion matrices for the conditions in experiment 2. Matrices for the higher SNR are shown in the left two columns, those for the lower SNR in the right columns. The columns indicate whether LFS was present in the stimulus or not and rows represent the individual HF cues. The color shading represents the identification rates (black indicates perfect identification). The label on the left side of the matrices denotes the vowels that were presented to the listeners, the label on the upper side of the matrix denotes the vowels that were identified by the listeners. The numbers in the matrices correspond to the percentage of this certain confusion.
(PDF)
