# Supplementary figures and images for: The Influence of High-Frequency Envelope Information on Low-Frequency Vowel Identification in Noise
Source: PLoS One. 2016 Jan 5;11(1):e0145610. doi: 10.1371/journal.pone.0145610 (PMC4701218; doi:10.1371/journal.pone.0145610)

no HF cue band

 $E_{\text{flat}}$  $E_{16}$ 

HFIC

 $\text{HFE}_{16}$ 

HFS

LPC

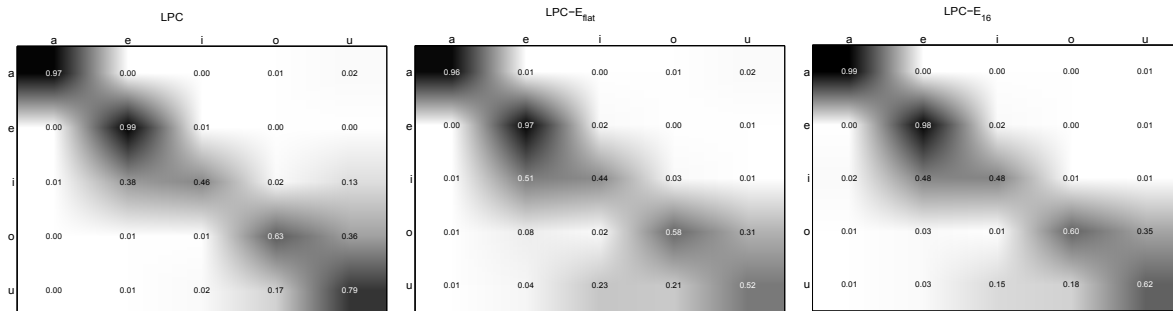

LFS

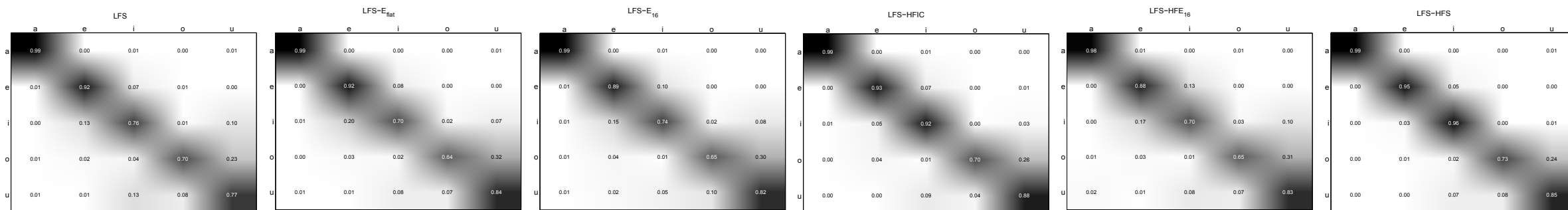

SNR = -14 dB

LPC

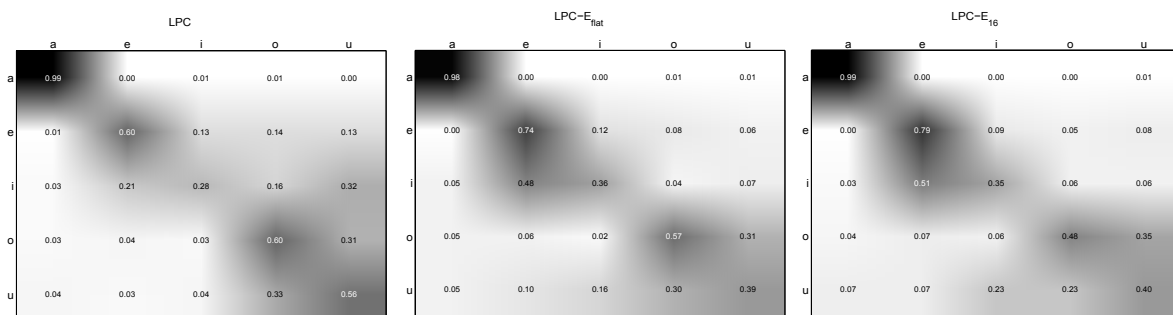

LFS

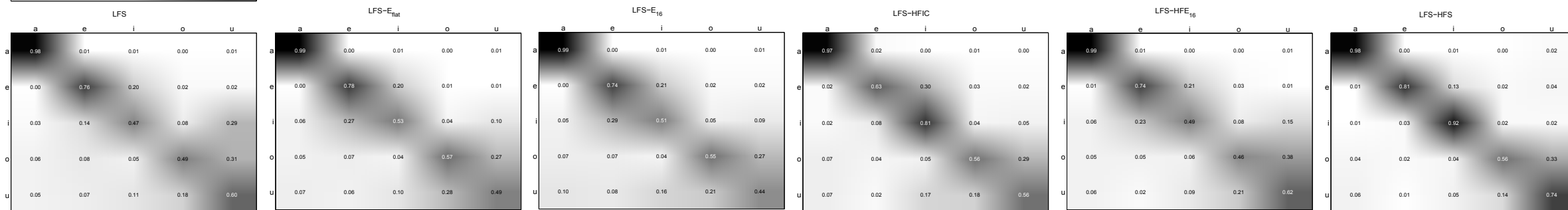

SNR = -18 dB

Supplement: S1 Fig — Matrices for the higher SNR are shown in the first two rows, those for the lower SNR in the last two rows. The rows indicate if LPC or LFS was used as a low-frequency part of the stimulus, columns indicate the type of high-frequency cue band that was presented. The color shading represents the identification rates. Black indicates perfect identification and white indicates no correct identification. The label on the left side of the matrix denotes those vowels that were presented to the listeners, the label on the upper side of the matrix denotes the vowels that were identified by the listeners. The numbers in the matrices correspond to the percentage of this certain confusion. (PDF) [file pone.0145610.s001.pdf]
