# Supplementary material for: The Influence of High-Frequency Envelope Information on Low-Frequency Vowel Identification in Noise
Source: PLoS One. 2016 Jan 5;11(1):e0145610. doi: 10.1371/journal.pone.0145610 (PMC4701218; doi:10.1371/journal.pone.0145610)
Supplement: S2 Fig — Matrices for the higher SNR are shown in the left two columns, those for the lower SNR in the right columns. The columns indicate whether LFS was present in the stimulus or not and rows represent the individual HF cues. The color shading represents the identification rates (black indicates perfect identification). The label on the left side of the matrices denotes the vowels that were presented to the listeners, the label on the upper side of the matrix denotes the vowels that were identified by the listeners. The numbers in the matrices correspond to the percentage of this certain confusion. (PDF) [file pone.0145610.s002.pdf]

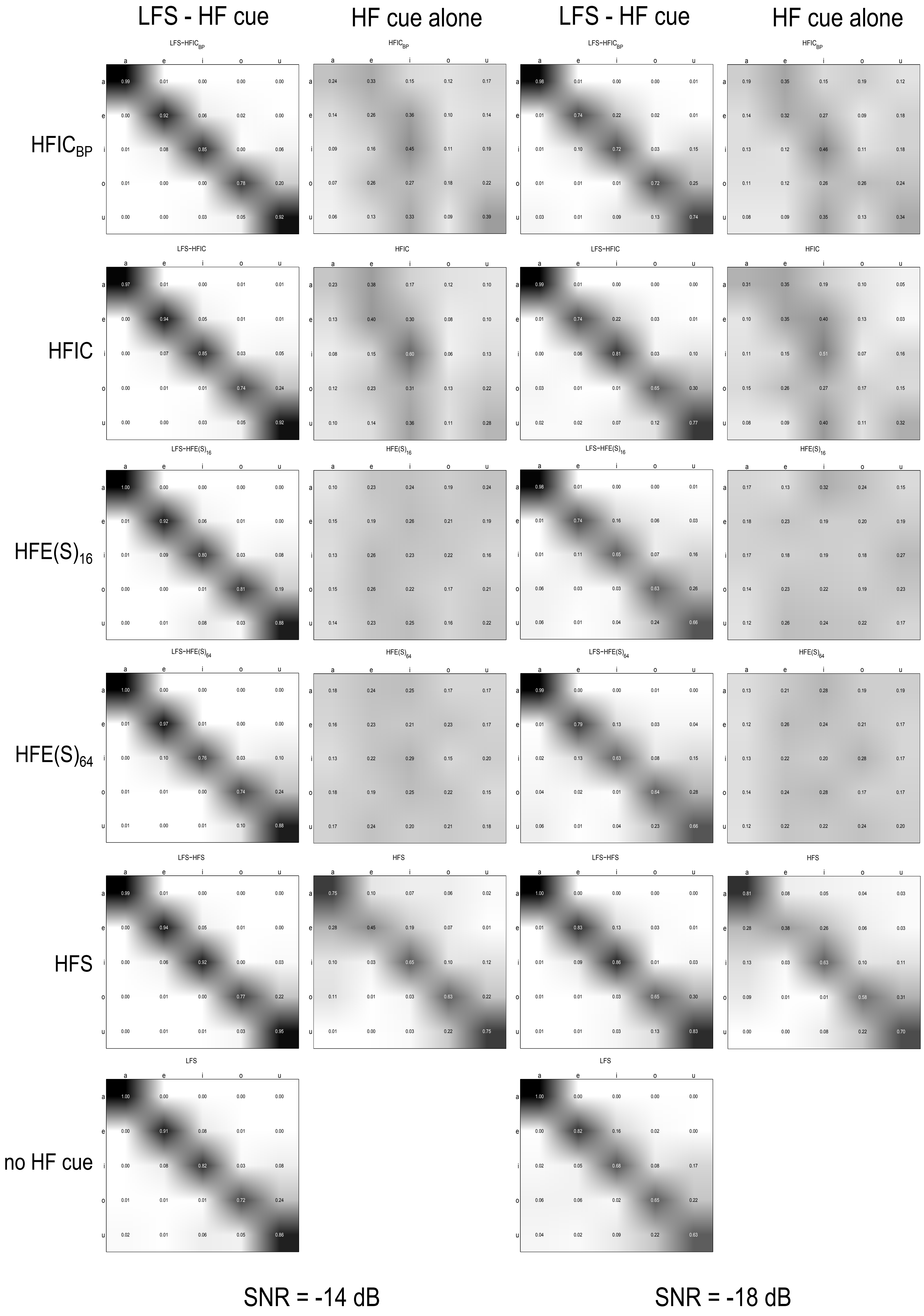

LFS - HF cue
HF cue alone
LFS - HF cue
HF cue alone
HFIC_BP
HFIC
HFE(S)16
HFE(S)64
HFS
no HF cue
LFS-HFIC_BP
HFIC_BP
LFS-HFIC
HFIC
LFS-HFE(S)16
HFE(S)16
LFS-HFE(S)64
HFE(S)64
LFS-HFS
HFS
LFS
a
e
i
o
u
SNR = -14 dB
SNR = -18 dB
